# Astrocytopathy Is Associated with CA1 Synaptic Dysfunction in a Mouse Model of Down Syndrome

**DOI:** 10.3390/cells14171332

**Published:** 2025-08-28

**Authors:** Álvaro Fernández-Blanco, Candela González-Arias, Cesar Sierra, Alfonsa Zamora-Moratalla, Gertrudis Perea, Mara Dierssen

**Affiliations:** 1 Center for Genomic Regulation (CRG), The Barcelona Institute for Science and Technology, 08003 Barcelona, Spain; alvaro.fernandez@crg.eu (Á.F.-B.); cesar.sierra@epfl.ch (C.S.); alfonsa.zamora@crg.eu (A.Z.-M.); 2 Department of Functional and Systems Neurobiology, Cajal Institute, CSIC, 28006 Madrid, Spain; candela.gonzalez@cajal.csic.es (C.G.-A.); gperea@cajal.csic.es (G.P.); 3 University Pompeu Fabra (UPF), 08005 Barcelona, Spain; 4 Centro de Investigación Biomédica en Red de Enfermedades Raras, 28029 Barcelona, Spain

**Keywords:** astrocytes, Down syndrome, astrocytopathy, hippocampus, astrocyte–neuron communication, intellectual disability

## Abstract

Brain pathophysiology in Down syndrome (DS), the most common genetic cause of intellectual disability, has traditionally been considered a consequence of neuronal dysfunction. However, although it is well documented that astrocytes play a critical role in brain homeostasis, synaptic regulation, and neuronal support, and their malfunction has been associated with the onset and progression of different neurological disorders, only a few studies have addressed whether astrocyte dysfunction can contribute to the DS pathophysiology. Astrocytes are increased in number and size, and show increased levels of expression of astroglial markers like S100β and GFAP. In this study, we detected a region-specific increase in astrocyte population in CA1 and, to a lesser extent, in the dentate gyrus. Single-nucleus transcriptomic profiling identified markers associated with reactive astroglia, synaptic transmission, and neuroinflammation in trisomic astrocytes. Functional analysis revealed abnormal Ca^2+^ oscillations in trisomic astrocytes and impaired astrocyte-to-neuron communication in CA1, the most affected subregion, leading to astrocyte-mediated excitatory synaptic depression. Our findings demonstrate that astrocytes play an active and critical role in the pathophysiology of DS, not only as reactive responders to neuronal injury but as key contributors to the disease process itself. This astrocytic dysfunction presents a region-specific distribution within the hippocampus, suggesting localized vulnerability and complex glial involvement in DS-related neuropathology.

## 1. Introduction

Astrocytes play critical roles in maintaining brain homeostasis, regulating synaptic activity [1,2], and providing metabolic support to neurons [3,4]. Growing evidence suggests that astrocytic dysfunction contributes to the onset and progression of different neurodevelopmental and cognitive disorders [5]. Among these alterations, changes in the number, size, and expression of astroglial proteins, as well as transcriptomic changes, have been widely described [6,7,8,9,10,11]. Down syndrome (DS) is a complex genetic disorder caused by the presence of an extra copy of chromosome 21. However, even though astroglial cells display pathological alterations across the lifespan of individuals with DS, the role of astrocytes in the pathophysiology of DS remains unknown.

Studies utilizing postmortem brain tissue from individuals with DS have shown that astrocytes are more numerous, larger, and express increased levels of astroglial markers (S100β, GFAP) [10,12]. In the hippocampus, DS fetuses have a greater fraction of cells with an astrocytic phenotype [13], indicating a neurogenic-to-gliogenic shift. This shift was confirmed in induced pluripotent stem cells (iPSCs) from monozygotic twins discordant for trisomy 21, in which trisomic cells showed increased expression of GFAP, S100β, and Vimentin [14]. Remarkably, S100β overexpression has been linked not only with impaired Ca^2+^ oscillations but also with reduced neuronal excitability in DS patient-derived iPSCs [15]. In addition to human studies, mouse models of DS have shown astroglial hypertrophy already in young trisomic mice, and increased number of astrocytes and higher expression of GFAP in the hippocampus of aged Ts65Dn mice [6,16]. Altogether, these results suggest that, in DS, astrocytopathy appears early during neurodevelopment and persists into adulthood. Yet, implications of a sustained astrocytopathy for neuronal communication and brain function are poorly understood.

We selected for our study the Ts65Dn mouse model, as it recapitulates many of the features found in DS. It includes trisomy for 125 human protein-coding orthologs, but the limitation is that trisomic mice also carry a triplication of 43 coding genes which are non-orthologous to HSA21. This can complicate the interpretation of genotype–phenotype relationships and potentially mask or amplify certain DS-related phenotypes. Even so, Ts65Dn mice recapitulate most of the results shown in humans [17], and other models have limitations in husbandry, including difficulties in breeding and maintaining large colonies, or present increased prevalence of hydrocephalus [18].

In the hippocampus of young-adult Ts65Dn male mice, we detected a general increase in astrocyte somatic volume and elevated expression of S100β. The astrocyte cell density was not modified, except in CA1, where it was increased. We also detected aberrant Ca^2+^ oscillations, and altered neuronal synaptic activity upon selective activation of astrocytes by chemogenetics, indicating a functional impact of astrocyte dysfunction. Single-nuclei RNAseq from control and Ts65Dn astrocytes revealed transcriptional changes related to cell proliferation, regulation of signaling, and synapse organization that may explain the structural and functional abnormalities observed.

Our results indicate that region-specific alterations in trisomic astrocytes might affect how astrocytes and neurons communicate in the Ts65Dn hippocampus and might contribute to cognitive impairments shown in Ts65Dn mice.

## 2. Materials and Methods

### 2.1. Animals

Ts(17^16^)65Dn/J (Ts65Dn; strain 005252) mice were obtained through crossings of a B6EiC3Sn a/A-Ts(17^16^)65Dn (Ts65Dn) female with B6C3F1/J males purchased from The Jackson Laboratory (Bar Harbor, ME, USA). Genotyping was performed by amplifying genomic DNA obtained from the mice tail according to the Jackson Laboratory indications. Mice had access to food and water ad libitum in controlled laboratory conditions, with temperature maintained at 22 ± 1 °C and humidity at 55 ± 10%, on a 12 h light/dark cycle (lights off 20:00 h). Mice were housed in numbers of two to four littermates. The colony of Ts65Dn mice was maintained in the Animal Facility of the Barcelona Biomedical Research Park (PRBB, Barcelona, Spain). Experiments were performed according to Directive 63/2010 and Member States’ implementation and followed the “Three Rs” principle of replacement, reduction, and refinement. The investigation was conducted in accordance with the Standards for Use of Laboratory Animals No. A5388-01 (NIH) and local (Law 32/2007), and European regulations and protocols were approved by the Ethics Committee of Parc de Recerca Biomèdica (Comité Ético de Experimentación Animal del PRBB (CEEAPRBB); authorization number MDS 0040P2). A/ES/05/I-13 and A/ES/05/14 grant the CRG permission to work with genetically modified organisms.

All the experiments were conducted in male Ts65Dn mice. All mice were aged two months ± 2 weeks at the time of the experiment.

We used male Ts65Dn for different relevant reasons. In the first place, to minimize the number of animals required to conduct the study, adhering to ethical considerations in animal research. Additionally, it enabled us to compare our studies with the existing literature in the field, which predominantly used male mice. In second place, by conducting this study in male mice, we minimized the experimental variability arising from hormonal fluctuations, as female sex hormones significantly influence astrocyte function, gene expression, and Ca^2+^ dynamics [19,20,21,22]. Therefore, we ensured that observed astrocyte dysfunction was directly attributable to trisomy rather than sex-specific differences. We acknowledge the importance of studying both sexes to better understand whether trisomic astrocyte dysfunction in the Ts65Dn hippocampus is contributed by sex differences. However, we believe that this initial exploratory study will serve as a foundation for future investigations that can delve into sex-specific differences of astrocyte dysfunction in DS.

### 2.2. Stereological Estimates

#### Tissue Preparation and Immunohistochemistry

For each experimental group, 40 μm coronal consecutive brain sections were obtained employing a vibratome (LeicaVT1200S, Wetzlar, Germany). The first section was identified at the stereotaxic coordinate bregma −1.34 mm. This region was selected because the somatic layers of the hippocampus are clearly distinguishable. Previous studies in the laboratory showed that taking every 6th did not show significant differences in the coefficient of variation (CV) using an ANOVA one-way test. Therefore 6 sections per mice of the dorsal hippocampus were taken into consideration for stereological estimates according to stereotaxic coordinates, namely bregma, −1.34 to −2.34 mm, regarding the mouse brain atlas [23], with the aid of a bright-field microscope (Zeiss Cell Observer HS, Jena, Germany).

Brain sections were washed with PBS (3 × 10′). Then, sections were permeabilized with 0.5% Triton X-100 (Sigma-Aldrich, St. Louis, MO, USA) in PBS (PBS-T 0.5%) (3 × 15′) and blocked with 10% of Normal Goat Serum (NGS) for two hours at room temperature. Sections were incubated in PBS-T 0.5% and NGS 5% with the primary antibodies overnight at 4 °C (mouse anti-NeuN 1:500, Merck-Millipore, #MAB377, Darmstadt, Germany; and rabbit anti-S100β 1:1000, Synaptic systems, #287003, Göttingen, Germany). Slices were washed with PBS-T 0.5% (3 × 15′) and incubated with the secondary antibodies (goat anti-mouse Alexa Fluor 555 for NeuN (1:500, Thermo Fisher Scientific, Waltham, MA, USA, #A11001) and goat anti-rabbit 488 for S100β (1:500, Thermo Fisher Scientific, Waltham, MA, USA, #A-11008) in the incubation buffer (PBS-T 0.5% + NGS 5%) for two hours at room temperature, protected from light. Finally, samples were washed with PBS-T 0.5% (3 × 15′), followed by PBS washing (3 × 10′) to remove the detergent, and sections were mounted onto a pre-cleaned glass slide with Mowiol mounting medium and coverslipped. Prior to immunostaining all the samples, an optimization of the primary antibodies and PBS-T conditions was performed with sections not needed for the estimations but sectioned with the same conditions. Serial dilutions of primary antibodies ranging from 1:100 to 1:1000 were prepared while maintaining the secondary antibody-concentration constant (1:500). Using confocal microscopy, the best primary antibody concentration was selected, taking into account the achievement of low background noise and the signal level obtained with the same laser configuration.

### 2.3. Stereological Estimates

Stereological estimates were performed using a Leica DMI6000B inverted microscope (Leica Microsystems, Wetzlar, Germany) equipped with a motorized stage, a microcator, and a digital camera connected to a PC that was used to obtain microscopic captures. Images were analyzed with the newCAST software (Version: 5.3.0.1562; Visiopharm Integrator System). All the estimations were performed single-blinded to avoid researcher bias.

### 2.4. Tissue Sampling and Stereological Methods

Mice were sacrificed with CO_2_ and transcardially perfused with ice-cold PBS, followed by 4% PFA in PBS (pH 7.4). Brains were extracted and post-fixed in 4% PFA at 4 °C overnight. Brains were then transferred to PBS, and 40 μm coronal consecutive brain sections were obtained using a vibratome (Leica VT1200S, Leica Microsystems, Wetzlar, Germany), collected in PBS, and stored in cryoprotective solution (40% PBS, 30% glycerol, and 30% polyethylene glycol) for long-term storage. Every 6th section was collected using the principle of systematic uniform random sampling. In total, six sections per mice were selected from bregma, −1.74 to −3.24 mm [23], with the aid of a bright-field microscope (Zeiss Cell Observer HS, Jena, Germany). We next followed the immunohistochemistry protocol previously described to stain and visualize astrocytes and neurons.

In order to precisely quantify the number of neurons and astrocytes in the different regions of the dorsal hippocampus, we used stereology, a technique that utilizes stringent sampling methods to obtain three-dimensional information about the entire tissue that is unbiased. Although 2D morphometry can provide quantitative information about the tissue sections being examined, it also makes several assumptions about the tissue, all of which are sources of bias, and can thus only be used in specific cases. Given the high neuronal density in CA1 and in the DG, stereological methods such as the optical prevent double-counting and precisely correct for the tridimensional volume of the region of study.

Prior to starting any stereological quantification, a quality check was performed in every section based on our expectations for global hippocampal architecture and specific immunostaining patterns. All the estimations were performed in one hemisphere, randomly chosen. Stereological estimates were performed in concrete ROIs of the hippocampus. Regions of interest were manually delineated according to the Franklin and Paxinos mouse brain atlas and the immunohistochemistry labeling method (see below). For astrocytic and neuronal populations, a 63× Oil 1.4 numerical aperture (NA) lens objective was required to resolve fine details in order to be able to (i) differentiate neurons inside a high density of cells (i.e., CA1 and DG) and (ii) clearly distinguish between somatic and astrocytic processes when moving through the *z*-axis.

### 2.5. Regional Volume Estimation of Hippocampal Subregions

Volume estimation of the selected ROIs (CA1, CA3, and DG somatic and dendritic layers) was calculated according to the Cavalieri principle, as it provides an estimate of the volume of any object when it is divided into parallel cross-sections. ROIs were selected according to anatomic boundaries following Franklin and Paxinos mouse brain atlas [23]. At least 200 points of known area were required in each ROI to obtain accurate volume estimations. For this reason, a specific point configuration was configured to ensure that more than 200 points in total were counted for every ROI across all the selected slices. For the Cavalieri estimation, the area of each ROI was 100% covered.

Volume estimation for the whole ROI and for every slice was calculated according to this formula:

Volume = T × a(p) × Σ P

where

T = spacing between sections;
a(p) = area per point given by each specific configuration;
Σ P = sum of points inside the ROI.

### 2.6. Quantification of Neurons and Astrocytes

Optical Disector (OD) uses thick sections and a microcator that allows one to focus on the *z*-axis of the section to count cells directly as they appear in an unbiased tridimensional counting frame (CF). For every region of interest, the percentage of the total sampling area was adjusted to obtain from 21 to 24 CFs. This range was established for two reasons: (i) fixing the same sampling area for every ROI can lead to biased results, as hippocampal subregions vary across the anteroposterior axis; and (ii) this CF range covers a representative area of each structure and is superior to the values found in the literature, thus ensuring a reliable estimation [24]. Real thickness of the planar sections was calculated for every section to avoid biased assumptions and adjust the tridimensional CF accordingly, as tissue is usually shrunken after histological fixation. CFs were uniform and randomly sampled across each ROI. CF zero position was set when the first cell became clear to vision, meaning the top view of the histological preparation. Cells were only counted if the nucleus was clearly visible inside the disector along the *z*-axis section or touching the inclusion lines (right and upper border); otherwise, neurons or astrocytes were discarded.

Zero μm position was set when the first cell became clear to vision, e.g., the top view of the histological preparation. Cells were only counted if the nucleus was clearly visible inside the disector along the *z*-axis section or touching the inclusion lines (right and upper border); otherwise, neurons were discarded. Each time that the upper right corner of the CF was inside the delineated ROI, a corner point (CP) mark was introduced to the counting, meaning that the CF was hitting the reference tissue. Once a CF was quantified, the microscope returned to the zero position, and the CF went to the next sampling position. To avoid potential double counting, the optical plane was moved throughout the whole thickness of the preparation section to ensure correct cell identification. Cell densities were calculated according to the Nv formula [25]:

$$Nv=\frac{\bar{{tQ}^{-}}}{BA}\cdot \frac{\sum Q^{-}}{h\cdot \left(\frac{a}{p}\right)\cdot \sum Q^{-}}$$

where

tQ− is the number-weighted mean section thickness;
BA is the Block Advance, the cut thickness of the section on a calibrated cutting device;
h is the disector height;
for (a/*p*), a is the area of the CF, and *p* is the number of points associated with the frame (1 − using the upper right corner of the CF);
Σ P is the sum of corner points hitting reference tissue.

The total number of cells (N) was estimated by the product of the cell density (Nv) and the volume (Vref) obtained from Cavalieri estimations:

N = V_ref_ × N_v_

CF dimensions were adjusted based on the lens objective used and the surface of a specific area to cover. For the somatic layers of the neuronal population, the frame was adjusted to cover 5% of the area of the field of view given for the 63× Oil objective.

### 2.7. Estimation of Astroglial Volume

In order to estimate astroglia volume, we utilized the S100β marker since it is mainly expressed in the astrocyte soma. We followed the immunohistochemistry protocol previously described to stain S100β with rabbit anti-S100β (1:1000, Synaptic systems; #287003) and visualize with goat anti-rabbit Alexa-555 (1:500, Thermo Fisher Scientific, Waltham, MA, USA, #A-32732). Astrocyte volume was estimated based on the spatial extent of S100β immunolabeling, and thus reflects the territory occupied by marker-positive processes above detection threshold. This approach does not provide absolute volumetric measurements but rather relative comparisons of detectable astrocytic domain size. Confocal fluorescence images were acquired at 20× magnification on a Leica TCS SP5 inverted scanning laser microscope, creating a composite image of the entire dorsal hippocampus at 16 bits. Tissue was only exposed to the lasers during the moment of image acquisition to prevent photobleaching. To minimize quenching of fluorescence, z-stacks were rapidly scanned at 1 µm increments. After image acquisition, stitched images of the dorsal hippocampus were converted to binary masks using ImageJ (version 1.52p) (Supplementary Figure S2A,B). To estimate the size of astroglial somas, the somatic area was semi-automatically identified in every plane, and the volume was calculated using the Cavalieri estimator (Supplementary Figure S2C–E):

Volume = ∑(A) × n × T

where

A = area of an astrocyte in a single plane (in µm^2^);
n = number of planes;
T = spacing between sections (in µm).

### 2.8. Quantification of S100β Fluorescence Intensity

In order to quantify the fluorescence intensity of S100β in euploid and trisomic astrocytes, we followed the immunohistochemistry protocol previously described to stain S100β with rabbit anti-S100β (1:1000, Synaptic systems; #287003) and then visualized with goat anti-rabbit Alexa-555 (1:500, Thermo Fisher Scientific, Waltham, MA, USA, #A-32732). Next, confocal fluorescence images were acquired at 20× magnification on a Leica TCS SP5 inverted scanning laser microscope, creating a composite image of the entire dorsal hippocampus at 16 bits. Confocal acquisition settings were maintained constant for all the samples, and all images were taken on the same day. The mean intensity of astroglial somas was performed by manually delineating the somas in accordance with the S100β signal using ImageJ. Signal background was subtracted for every region and image.

### 2.9. Astrocyte Tagging and Manipulation Techniques

#### Viral Constructs

In order to identify and manipulate astrocytes, we used a construct that expressed the excitatory DREADD hM3D (Gq)-mCherry under the GFAP promoter. AAV5-GFAP-hM3D (Gq)-mCherry was a gift from Bryan Roth (Addgene Viral prep # 50478-AAV5; RRID:Addgene_50478). As a control, we used AAV5-GFAP104-mCherry (gift from Edward Boyden; Addgene viral prep #58909-AAV5). We also used a construct to express a genetically encoded calcium indicator (cyto-GCaMP6f) under the gfaABC1 promoter, a shortened 681 bp GFAP promoter. AAV5-gfaABC1D-cyto-GCaMP6f was a gift from Baljit Khakh (Addgene viral prep #52925-AAV5; RRID: Addgene 52925-AAV5) [26]. Viral titers were 1.4 × 10^13^ genome copy (GC)/mL for AAV5-GFAP-hM3D (Gq)-mCherry, 1.4 × 10^13^ GC/mL for GFAP104-mCherry, and 1.3 × 10^13^ GC/mL for gfaABC1D-cyto-GCaMP6f. For ex vivo calcium imaging studies, a 1:2 mixture of both AAV5-GFAP-hM3D (Gq)-mCherry and AAV5-gfaABC1Dcyto- GCaMP6f was used.

### 2.10. Stereotaxic Surgery Injection

Intracerebral injections in CA1 were performed bilaterally at bregma −2.5 mm AP, +/−2.0 mm ML, −1.5 mm DV, with the aid of a stereotaxic apparatus (Stoelting 51730, Wood Dale, IL, USA). For electrophysiology ex vivo studies, mice were 5–7 weeks at the time of surgery. Mice were anesthetized using ketamidol (7.5 mg/kg) and medetomidine (0.2 mg/kg). Fur was shaved from the incision site. Skin was wiped with 70%ethanol, and small incisions were made along the midline to expose bregma and injection sites. Craniotomies were performed using a 0.45 mm diameter stereotaxic drill (RWD Life Science, East Hartford, CO, USA, model 78001). Viral vectors were injected at 60 nl/min through a 33-gauge cannula (Plastics One, C235I/Spc) attached to a Hamilton microsyringe (1701N; Hamilton) connected to a syringe pump (PHD 2000, Harvard Apparatus, Holliston, MA, USA) for 10 min. For ex vivo calcium imaging studies, a volume of 600 nl/hemisphere of a 1:2 mixture of both AAV5-GFAP-hM3D (Gq)-mCherry and AAV5-gfaABC1D-cyto-GCaMP6f was used. Cannulas remained 10 min after injection to allow virus diffusion and were slowly withdrawn for 5 min. Skin was sutured, and mice were treated with 0.03 mg/kg buprenorphine as an analgesic. Mice were recovered from anesthesia by Atipamezole (1 mg/kg) and maintained on a heating pad until fully recovered. Mice were allowed to recover for 3 weeks before experimentation to allow for construct expression. After sacrifice, all injection sites were verified histologically. Only those mice in which virus expression was restricted to the dorsal CA1 were included for analysis.

### 2.11. Calcium Imaging

To characterize spontaneous and evoked Ca^2+^ oscillations, coronal hippocampal slices (300 μm) were obtained from 8–10-week Ts65Dn and WT male littermates injected with AAV5-GFAP-hM3D (Gq)-mCherry and AAV5-gfaABC1D-cyto-GCaMP6f. Animals were sacrificed, and brains were quickly removed and sliced using a vibratome (Leica VT1200S, Leica Microsystems, Wetzlar, Germany) in ice-cold oxygenated low-Ca^2+^ ACSF (126 mM NaCl, 2.6 mM KCl, 26 mM NaHCO_3_, 1.25 mM NaH_2_PO_4_, 10 mM Glucose, 1 mM MgCl_2_, and 0.625 mM CaCl_2_). Slices were then incubated for 1 h with oxygenated normal Ca^2+^ ACSF (126 mM NaCl, 2.6 mM KCl, 26 mM NaHCO_3_, 1.25 mM NaH_2_PO_4_, 10 mM Glucose, 1 mM MgCl_2_, and 2 mM CaCl_2_). Individual slices were transferred to a recording chamber in an Olympus BX51WI upright microscope at room temperature (20–22 °C) in the presence of picrotoxin (50 μM). Images were acquired at 1 Hz for 150 s, having 6–15 astrocytes in the field of view of the camera using epifluorescence microscope (Olympus BX51 WI upright). In the first 120 s, the spontaneous astrocyte Ca^2+^ activity was measured. Between second 118 and 120, a 2 s pulse (1 bar) was applied using a patch pipette to locally apply CNO (1 mM) into the field of view. Then, astrocyte Ca^2+^ activity post-CNO administration was measured from 120 s to 150 s. Co-localization of GCaMP6f with hM3D (Gq)-mCherry was checked after the recording. Image acquisition and image analysis was performed using a cellSens software (Version 1.4, Olympus, Hachioji, Japan). Ca^2+^ oscillations were recorded from the cell body. Background was subtracted for every image. Data were then fed into an in-house code kindly provided by Perea lab that identified Ca^2+^ events when the signal showed maximum values above 2 times the standard deviation of the previous steady signal [27]. Ca^2+^ variations were estimated as changes in the fluorescence signal (ΔF) over the baseline (F_0_). Mean event frequency and amplitude values for each experimental group were obtained by averaging the mean amplitude and frequency over a 2 min period. Astrocytes with no oscillations were not included in the analysis. Frames with movement artifacts were excluded for the analysis.

### 2.12. Minimal Stimulation Protocol

We used a minimal stimulation protocol to investigate whether astrocyte-to-neuronal communication is impaired in Ts65Dn mice. Coronal hippocampal slices (300 µm) were made from 8–10-week WT and Ts65Dn littermates previously injected with AAV_5_-GFAP-hM3D (Gq)-mCherry in CA1. Animals were decapitated, and brains were quickly removed and sliced using a vibratome (Leica VT1200S, Leica Microsystems, Wetzlar, Germany) in an ice-cold (4 °C) NMDG-HEPES solution (92 mM NMDG, 2.5 mM KCl, 30 mM NaHCO_3_, 1.2 mM NaH_2_PO_4_, 20 mM HEPES, 5 mM Na-ascorbate, 2 mM thiourea, 3 mM Na-pyruvate, 25 mM Glucose, 10 mM MgSO_4_ 7H_2_O, and 0.5 mM CaCl_2_ 2H_2_O) [28]. Subsequently, slices were placed in the same NMDG-HEPES solution at 32–34 °C for 10 min. Slices were then incubated for 1 h with oxygenated normal Ca^2+^ACSF (124 mM NaCl, 2.69 mM KCl, 26 mM NaHCO_3_, 1.25 mM KH_2_PO_4_, 10 mM Glucose, 2 mM MgSO_4_, and 2 mM CaCl_2_). Individual slices were transferred to a recording chamber in an Olympus BX50WI upright microscope (Olympus Optical). Electrophysiological recordings were filtered at 1 kHz and acquired at a 10 kHz sampling rate with PC-ONE amplifier (Dagan Corporation, Axon Instruments, MN, USA) and digitized by a 1440 A DigiData interface board (Axon Instruments, Union City, CA, USA). Patch pipettes of borosilicate glass were pulled (Sutter P-1000, Sutter Instruments, Sycamore, IL, USA) and then filled with internal solution for whole-cell somatic voltage clamp recordings (135 mM K-gluconate, 10 mM KCl, 10 mM HEPES, 1 mM MgCl_2_, and 2 mM ATP-Na_2_). The membrane potential was held at −70 mV while fast and slow whole-cell capacitances were neutralized and series resistance was compensated (by around 70%). Before and after the trials, electrophysiological parameters were monitored. Throughout the experiment, a −5 mV pulse was used to keep track of the series and input resistances. When the series and input resistances, resting membrane potential, and stimulus artifact duration did not fluctuate by more than 20%, recordings were considered stable.

Synaptic responses in CA1 were evoked by stimulating the SC fibers with theta capillaries (2–5 µm tip diameter) filled with ACSF electrodes delivering pulses of 50 μs, at a duration of 0.5 Hz. Recordings were performed in the presence of picrotoxin (50 µM). Stimulus intensity was set to stimulate single or very few synapses and was kept constant during the experiment. Stimulus intensity was also adjusted to achieve a 50% failure and 50% success of CA1-evoked synaptic responses. Basal-evoked responses were measured for 5 min. At min 5, CNO was locally applied at 1 mM with a puff (2 s, 1 bar), and then activity was measured for 10 more min. The following synaptic parameters were examined: synaptic efficacy (mean peak amplitude of all responses, including failures), synaptic potency (mean peak amplitude of the successes), and probability of release (Pr, ratio between number of successes versus total number of stimuli). Synaptic parameters were evaluated before and after astrocyte stimulation with CNO. For statistical purposes, basal responses and responses 5 min after CNO stimulation were considered. Experiments were performed at room temperature (22 ± 2 °C).

### 2.13. Single-Nucleus RNA Sequencing Data Analysis

The single-cell RNA sequencing data analyzed in this study were generated as previously described [29]. Briefly, tissue was dissociated, NeuN negative neuronal nuclei were sorted using fluorescence-activated nuclear sorting (FANS), and libraries were prepared using the 10× Genomics Chromium Single Cell Kit Version 3 (10× Genomics Pleasanton, CA, USA) and sequenced on a NovaSeq 6000 S1 (Illumina, San Diego, CA, USA) to an average depth of approximately 20,000 reads per cell.

#### 2.13.1. 10× Data Pre-Processing

The readings were matched to the reference genome, including exons and introns, and transformed to mRNA molecule counts using the manufacturer’s Cellranger pipeline (CellRanger v3.0.1). We counted the number of genes for which at least one read was mapped for each nucleus, and then we discarded any nuclei with fewer than 200 or more than 2500 genes to eliminate low-quality nuclei and duplets, respectively. Genes found in fewer than six nuclei were discarded. To normalize for differences in coverage, expression values Ei,j for gene I in cell j were calculated by dividing UMI counts for gene I by the sum of UMI counts in nucleus j, multiplying by 10,000 to create TP10K (transcript per 10,000) values, and finally computing log2 (TP10K + 1) (using the NormalizeData function from the Seurat package v.2.3.4) [30].

#### 2.13.2. Batch Correction and Scaling Data Matrix

Because samples were processed in two different experiments, batch correction and data scaling were executed as previously described [29]. Briefly, Harmony was used on the normalized dataset, and then the data were scaled using the ScaleData function from Seurat [31]. The scaled data matrix was then used for dimensionality reduction and clustering.

#### 2.13.3. Dimensionality Reduction, Clustering and Visualization

Using the RunPCA method in Seurat (a wrapper for the irlba function), we computed the top 60 principal components using the scaled expression matrix restricted to the variable genes. UMAP (Uniform Manifold Approximation and Projection) used the scores from these principal components as input to downstream grouping and visualization (UMAP). The FindNeighbors and FindClusters functions in Seurat (resolution = 0.6) were used to cluster the data. After that, using UMAP, the clusters were visualized. Before integrating with the IntegrateData function, reference anchors between genotypes were found, and the combined data were analyzed using the same procedures.

SingleR (version 1.0.6) [32] was used for single-cell annotation based on the “MouseRNAseqData” dataset from the celldex package (version 1.0.0) and using the “label.main” to assign cell subtypes. Clusters at a resolution of 0.05 were annotated based on the most prevalent predicted cell subtypes. The annotation was further refined by mapping the most enriched genes for each cluster (identified using the FindAllMarkers function) to the cell types of the Linnarson mouse atlas [33].

### 2.14. Identification of Marker Genes Within Every Cluster

The FindAllMarkers function was used to find cluster-specific marker genes using a negative binomial distribution (DESeq2). A marker gene was defined as having a detectable expression in >20% of the cells from the related cluster and being >0.25 log-fold greater than the mean expression value in the other clusters. We were able to choose markers that were highly expressed within each cluster while still being restricted to genes unique to each individual cluster.

### 2.15. Identification of Differentially Expressed Genes Between WT and Ts65Dn

Within each cell type, WT and TS samples were compared for differential gene expressions using Seurat’s FindMarkers function. To be included in the analysis, the gene had to be expressed in at least 10% of the cells from one of the two groups for that cell type, and there had to be at least a 0.25-fold change in gene expression between genotypes. After correcting for multiple testing, only genes with a *p*-adjusted value < 0.001 were considered for downstream analyses.

### 2.16. Diffusion Map

Using the DiffusionMap function from the destiny package in R [34] (with k = 30 and a local sigma), the diffusion components were calculated using the cell embedding values in the top 15 principal components (generated either on the scaled expression matrix restricted to the variable genes in the 7-month-old mouse dataset or on the aligned canonical correlation analysis (CCA) subspace for the entire time-course data). For data visualization, we selected the top two diffusion components. This function allowed us to discover drivers of cell embedding at a global or subregion level using non-linear low-dimensional embeddings like UMAP or Diffusion Map.

### 2.17. Cellular Proportion

The proportional fraction of nuclei in each cell type was standardized to the total number of nuclei taken from each library to acquire insight into cell-type variations in the trisomic hippocampus. We used single-cell differential composition analysis (scDC) to bootstrap proportion estimates for our samples to see if any changes in cell-type proportion were statistically significant [35].

### 2.18. Gene Set Enrichment

The differential expression signatures from each cellular subtype were tested for enriched Gene Ontology processes, using a hypergeometric test (shinyGO), and corrected for 26 multiple hypotheses by false discovery rate (FDR). Processes with an adjusted *p*-value < 0.05 were reported as being significantly enriched. The complete list of genes present in the dataset was used as the universe for the hypergeometric test.

### 2.19. Statistical Analysis

When two conditions were compared, the Shapiro–Wilks test was conducted to check the normality of the data, and Fisher’s F test was used to assess the homogeneity of variances between groups. When data met the assumptions of parametric distribution, the results were analyzed by the unpaired Student’s *t*-test. Paired *t*-tests were employed to compare paired variables. The Mann–Whitney–Wilcoxon test was applied in cases where the data did not meet the requirements of normal distribution. Statistical analyses were two-tailed.

For comparison between more than two groups, two-way ANOVA with different levels was conducted, followed by Tukey’s HSD multiple comparison test. The Bartlett test was used to assess the homogeneity of variances between groups. If the data distribution was non-parametric, the Kruskal–Wallis test was used, followed by the Mann–Whitney–Wilcoxon test. All statistical analyses were two-tailed. The statistical test used is indicated in every figure. Differences in means were considered statistically significant at *p* < 0.05. Data analysis and statistics were performed using R studio (Version 1.1.463).

## 3. Results

### 3.1. Astrocytopathy Is Not Evenly Distributed Across Hippocampal Regions in Ts65Dn

**Figure 1:**
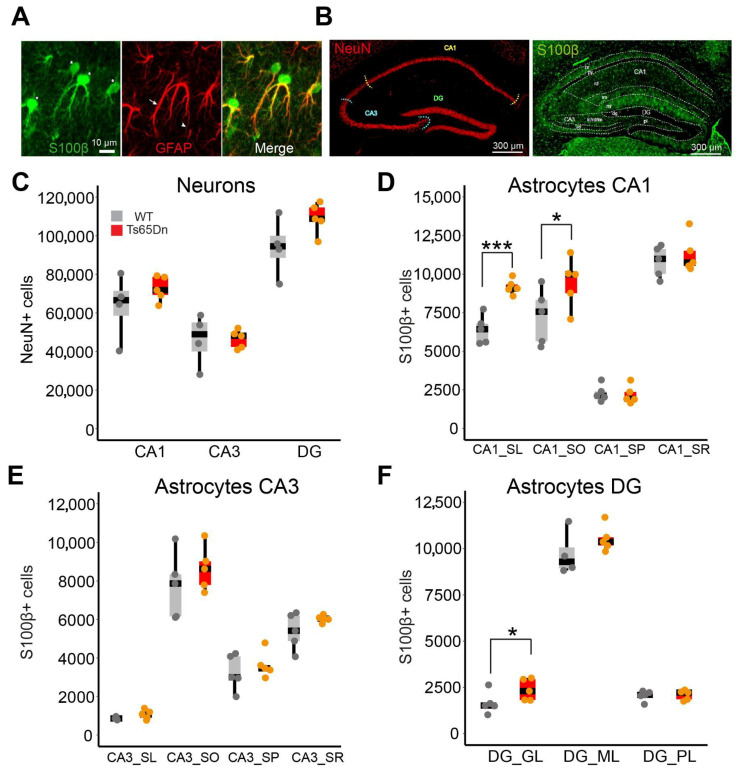
Subregion-specific astrocytopathy in Ts65Dn hippocampus. (**A**) Representative images of the different antibodies used against astroglial antigens. Left: S100β is mainly located in the astrocyte soma (asterisks). Middle: GFAP is mainly distributed in the astroglial processes (arrows and arrowheads). Right: Merged image. Scale bar = 10 µm. (**B**) Left: NeuN distribution in the dorsal hippocampus using the NeuN neuronal marker (in red). Right: S100β distribution in the dorsal hippocampus (in green). Scale bar = 300 µm. (**C**) Stereological estimates of NeuN+ numbers in the somatic regions of the hippocampus (CA1, CA3, and DG). Dots represent the mean of 6 different sections of individual mice (gray represents WT and orange Ts65Dn). (**D**–**F**) Boxplots showing the stereological estimations of S100β+ cells in (**D**) the CA1 somatic (CA1_SP) and dendritic layers (CA1_SL, CA1_SO, and CA1_SR). (**E**) The CA3 somatic (CA3_SP) and dendritic layers (CA3_SL, CA3_SO, and CA3_SR). (**F**) The DG somatic (DG_GL) and dendritic regions (DG_ML and DG_PL). WT = 4–5; Ts65Dn = 5. Every dot corresponds to the average of six sections. On the boxplots, the horizontal line indicates the median, the box indicates the first to third quartile of expression, and whiskers indicate 1.5 × the interquartile range. *** *p* < 0.001; * *p* < 0.05. Two-tailed *t*-test or Mann–Whitney depending on the normality of the data. SL = *stratum lacunosum*, SO = *stratum oriens*, SP = *stratum pyramidale*, SR = *stratum radiatum*, GL = granule layer, ML = molecular layer.

We quantified the number of neurons (NeuN+) and astrocytes (S100β+) in all subregions of the dorsal hippocampus in WT and Ts65Dn mice (Figure 1A,B) by using the systematic random optical dissector method (see Section 2). We selected S100β+ to quantify astrocytes because GFAP was mainly located in astroglial processes (Figure 1A). We also analyzed astrocyte cell volume and the S100β expression levels in both the somatic and dendritic layers of the dorsal hippocampus (see Section 2 and Supplementary Figure S2).

**Figure 2:**
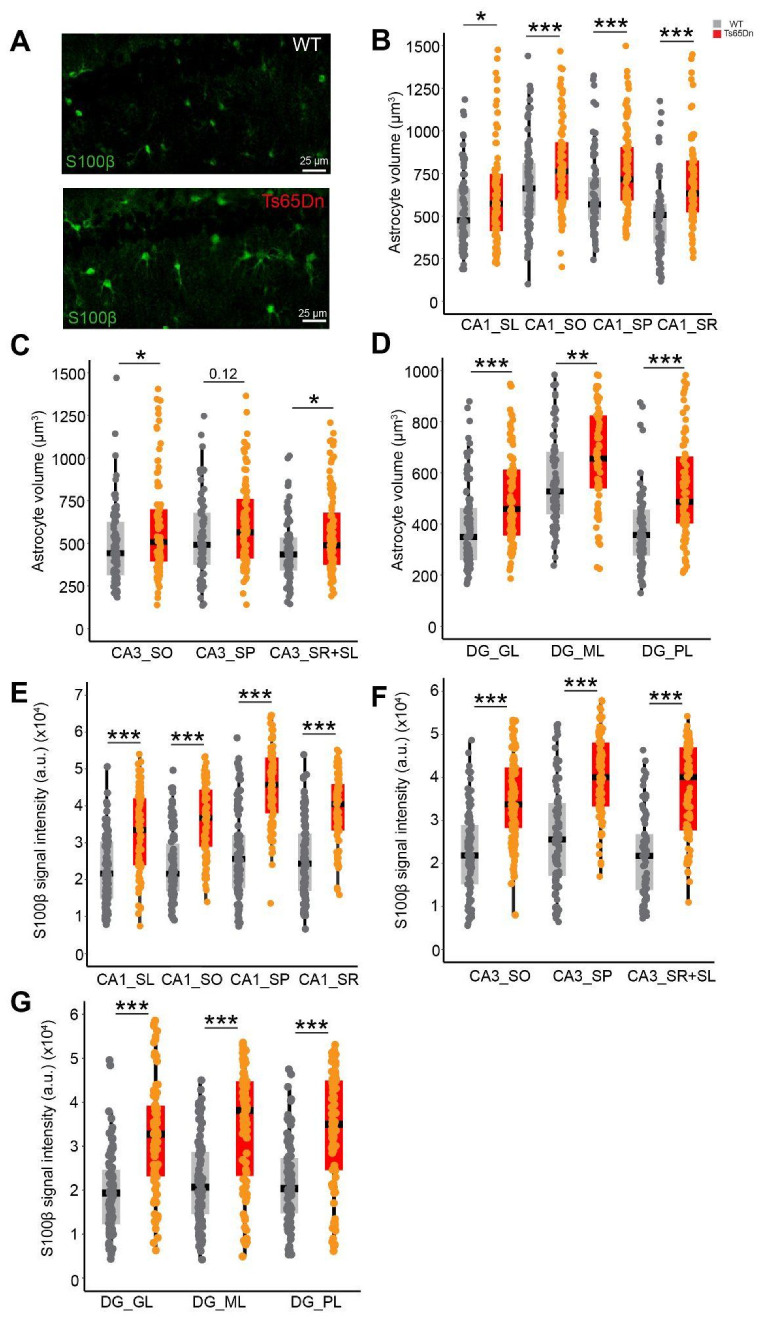
Increased astroglial somatic volume and S100β expression in Ts65Dn astrocytes. (**A**) Representative image of WT (above) and Ts65Dn (below) S100β expression in CA1. It is noticeable that astrocytes are larger in Ts65Dn. Scale bar = 25 µm. (**B**–**D**) Quantification of astroglial volume (in µm^3^) in (**B**) CA1 layers, (**C**) CA3 layers, and (**D**) DG layers. Gray dots indicate the estimations of individual WT mice (n = 61–106 astrocytes from 4 mice), while orange dots indicate the estimations of the individual Ts65Dn mice (n = 58–103 astrocytes from 4 mice). (**E**–**G**) Elevated S100β intensity signal in Ts65Dn astrocytes compared to euploid mice was detected in (**E**) CA1, (**F**) CA3, and (**G**) DG subregions. Gray dots indicate the estimations of individual WT mice (n = 81–94 astrocytes from 4 mice), while orange dots indicate the estimations of Ts65Dn (n = 81–97 astrocytes from 4 mice). On the boxplots, the horizontal line indicates the median, the box indicates the first to third quartile of expression, and whiskers indicate 1.5× the interquartile range. *** *p* < 0.001, ** *p* < 0.01, * *p* < 0.05, Mann–Whitney. SL = *stratum lacunosum*, SO = *stratum oriens*, SP = *stratum pyramidale*, SR = *stratum radiatum*, GL = granule layer, ML = molecular layer.

No differences were observed in the volume of the different hippocampal subregions (CA1, CA3, or the DG; Supplementary Figure S1) nor in the number or the density of NeuN+ cells between WT and Ts65Dn mice (two-tailed *t*-test; N.S.; Figure 1C and Figure S1A). However, the number of S100β+ cells was significantly higher in CA1 *stratum lacunosum* (two-tailed *t*-test; *p* < 0.001; *n* = 5 *mice per genotype*; Figure 1D), CA1 *stratum oriens* (two-tailed *t*-test; *p* < 0.05; *n* = 5 *mice per genotype*; Figure 1D), and the DG granule cell layer (two-tailed t-test; *p* < 0.05; *n* = 5 *mice per genotype*; Figure 1F) of trisomic compared to WT mice, along with increased astroglial density in CA1 *stratum lacunosum* (two-tailed *t*-test; *p* < 0.001; *n* = 5 *mice per genotype*; Supplementary Figure S1C) and in DG granule cell layer (two-tailed *t*-test; *p* < 0.05; *n* = 5 *mice per genotype*; Supplementary Figure S1G). No differences were detected in CA1 *stratum pyramidale*, *radiatum*, or DG *molecular* and *polymorphic layers* (two-tailed *t*-test; *p* = N.S. *n* = 5 *mice per genotype*; Figure 1F). No increase in S100β+ number or density was found in CA3 (two-tailed *t*-test; *p* = N.S. *n* = 5 *mice per genotype*; Figure 1E). Ts65Dn S100β+ cells had a larger soma than their euploid littermates in all the hippocampal regions, except the CA3 pyramidal layer (Mann–Whitney; *p* = 0.12; *n* = 61–106 *astrocytes from 4 WT mice,* 58–103 *astrocytes from* 4 *Ts65Dn mice*; Figure 2A–D). S100β expression levels were significantly higher in Ts65Dn than in WT astrocytes in all the hippocampal regions (Mann–Whitney; *p* < 0.001; *n* = 81–94 *astrocytes from* 4 *WT mice,* 81–97 *astrocytes from* 4 *Ts65Dn mice*; Figure 2E–G).

In summary, while CA1 and DG present increased number of somas and soma size in the astrocytes, CA3 shows a milder phenotype, with no significant astrocytopathy and a less marked increase in astrocyte soma size, but increased S100β levels, suggesting that while there may be some neuroinflammatory response, the structural and functional aspects of CA3 astrocytes might remain relatively intact.

### 3.2. Trisomic Astroglia Exhibit Abnormal Ca^2+^ Dynamics in CA1

**Figure 3:**
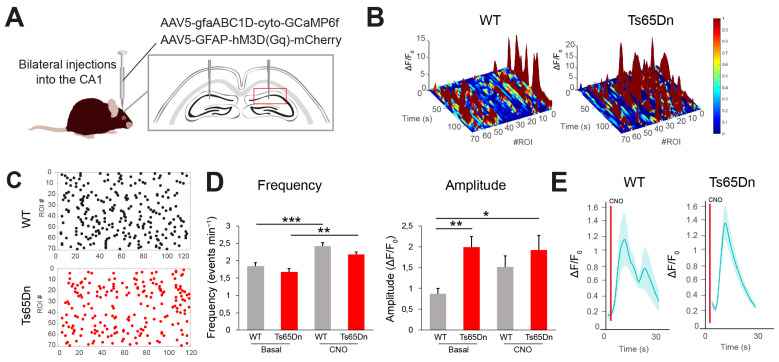
Ts65Dn astrocytes exhibit abnormal Ca^2+^ dynamics. (**A**) Experimental design: AAV_5_-GFAP-hM3D (Gq)-mCherry and AAV_5_-gfaABC1D-cyto-GCaMP6f were targeted bilaterally to the dorsal CA1 stratum radiatum region of WT and Ts65Dn mice. (**B**) Left: Changes in ΔF/Fo over time for multiple WT astrocytes in CA1 stratum radiatum in baseline conditions. Right: Changes in ΔF/Fo over time for multiple Ts65Dn astrocytes (WT = 70 astrocytes from 3 mice; Ts65Dn = 70 astrocytes from 3 mice). (**C**) Mapping of the different events for every astrocyte in WT (left) and Ts65Dn (right) mice. Each dot represents an event (WT = 253 events from 70 astrocytes; Ts65Dn = 205 events from 70 astrocytes). (**D**) Left: Basal and post-CNO Ca^2+^ oscillation frequency both in WT (gray) and Ts65Dn (red) astrocytes. Right: Basal and post-CNO Ca^2+^ oscillation amplitude in WT and Ts65Dn astrocytes (WT basal = 87 astrocytes from 3 mice; Ts65Dn basal = 109 astrocytes from 3 mice; WT post-CNO = 70 astrocytes from 3 mice; Ts65Dn post-CNO = 68 astrocytes from 3 mice). (**E**) Time course depicting the mean intracellular Ca^2+^ signal changes after the local application of CNO (1 mM). CNO application is indicated by a red line at second 5 (WT = 70 astrocytes from 3 mice; Ts65Dn = 68 astrocytes from 3 mice). Data are expressed as mean ± SEM. *** *p* < 0.001, ** *p* < 0.01, and * *p* < 0.05. Two-way ANOVA, with Tukey HSD as post hoc.

Astrocytopathy is usually associated with changes in astrocyte physiology, and several studies have reported changes in astrocyte Ca^2+^ oscillations, which are critical for astrocytic signaling, homeostasis, and communication with neurons [15]. In order to investigate whether trisomy had an impact in trisomic astrocyte physiology, we focused on the CA1 region that showed the most important changes in trisomic mice. We expressed a genetically encoded Ca^2+^ indicator (GCaMP6f) driven by the astrocyte-specific GfaABC1B promoter in CA1 astrocytes, along with hM3D (Gq)-mCherry in WT and Ts65Dn mice (Figure 3A). Gq-coupled designer receptors induce reversible and time-restricted Ca^2+^ oscillations in astrocytes when activated by clozapine N-oxide (CNO) [36,37].

First, we evaluated spontaneous Ca^2+^ oscillations in the presence of the GABA_A_ receptor antagonist picrotoxin (50 µM) in order to block inhibitory neurotransmission [1]. Ca^2+^ oscillations can only be measured in oscillating astrocytes in the CA1 stratum radiatum region. The basal-event frequency (e.g., the rate at which Ca^2+^ or oscillations occur in baseline conditions) was similar in WT and Ts65Dn astrocytes (Figure 3B–E). Nevertheless, the average basal amplitude of Ca^2+^ events (ΔF/F_0_) was significantly higher in Ts65Dn astrocytes (two-way ANOVA; genotype: F (1330) = 9.67, *p* = 0.002; post hoc Tukey HSD; *p* = 0.001; n = WT basal = 87 astrocytes from 3 mice, Ts65Dn basal = 109 astrocytes from 3 mice; Figure 3D; right panel, Supplementary Figure S3A,B). To assess intracellular Ca^2+^ oscillations, we locally applied a puff of CNO while examining the time course of Ca^2+^ responses. CNO application led to a rise in the intracellular Ca^2+^ signal in astrocytes (Figure 3D). Event frequency was significantly increased both in WT (two-way ANOVA; treatment: F (1330) = 31.07, *p* < 0.001; post hoc Tukey HSD; *p* < 0.001; n = WT post-CNO = 70 astrocytes from 3 mice, Ts65Dn post-CNO = 68 astrocytes from 3 mice; Figure 3D) and in Ts65Dn astrocytes (post hoc Tukey HSD; *p* = 0.0059; Figure 3D), but amplitude was not modified upon CNO application neither in WT nor Ts65Dn astrocytes (Figure 3D).

### 3.3. Activation of Ts65Dn Astrocytes Leads to Synaptic Depression

Ca^2+^ dynamics is essential for astrocyte-to-neuron communication [38,39,40]. As such, the increased amplitude of Ca^2+^ signals in trisomic mice may enhance the release of gliotransmitters [41,42], which can impact synaptic transmission.

**Figure 4:**
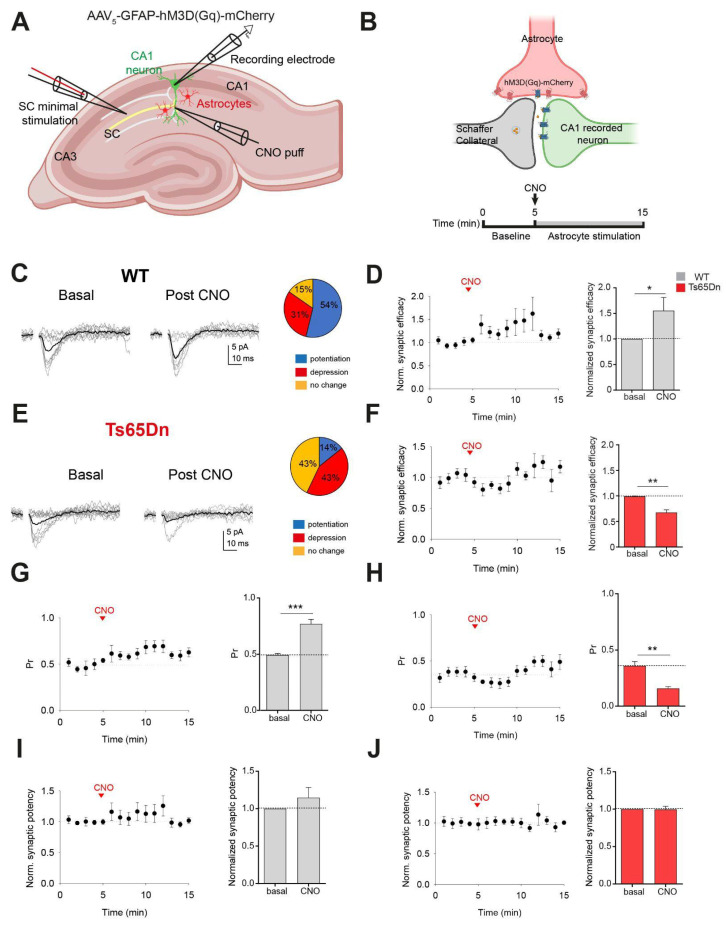
Trisomic astrocyte manipulation induced a short-term synaptic depression in single synapses. (**A**) Schematic drawing showing the minimal stimulation protocol. Schaffer collaterals (SCs) were stimulated by an electrode while recording a CA1 pyramidal neuron. Changes in synaptic transmission were evaluated upon activating hM3D (Gq)-expressing astrocytes by a local CNO puff. (**B**) Above: Schematic representation of the hM3D (Gq)-mCherry-expressing astrocyte terminal surrounding pre- and postsynaptic neurons. Below: Experimental timeline: evoked basal synaptic transmission is recorded during 5 min. At min 5, a CNO puff is applied and evoked synaptic responses are recorded for 10 min. (**C**,**E**) EPSCs failures and successes (in gray) in basal conditions (left) and after CNO (right), and synaptic efficacy (mean response, in black). Proportion of synapses that showed potentiation (blue), depression (red) or no changes (orange) in WT (**C**) and Ts65Dn (**E**) mice. (**D**,**F**) Left: Normalized synaptic efficacy changes over 15 min in WT mice (**D**) (n = 7 out of 13 astrocyte–neuron pairs from 5 mice) and in Ts65Dn mice (**F**) (n = 6 out of 14 astrocyte–neuron pairs from 4 mice). Right: Relative changes in normalized synaptic efficacy in basal conditions and after astrocytes stimulation in WT (**D**) and in Ts65Dn mice (**F**). (**G**) Probability of release (Pr) changes over 15 min (left) and relative changes in Pr in basal conditions and after astrocytes stimulation (right) in WT mice (n = 7 out of 13 astrocyte–neuron pairs from 5 mice) and in Ts65Dn (**H**) mice (n = 6 out of 14 astrocyte–neuron pairs from 4 mice). (**I**) Normalized synaptic potency changes over 15 min (left) and relative changes in the normalized synaptic potency in basal conditions and after astrocytes stimulation (right) in WT mice (n = 7 out of 13 astrocyte–neuron pairs from 5 mice) and in Ts65Dn (**J**) mice (n = 6 out of 14 astrocyte–neuron pairs from 4 mice). Data are expressed as mean ± SEM. *** *p* < 0.001, ** *p* < 0.01, and * *p* < 0.05. Paired *t*-test.

We investigated whether the excitatory synaptic transmission in trisomic CA3-CA1 synapses was modified upon astrocyte manipulation. We expressed the excitatory DREADD Gq fused to mCherry (AAV_5_-GFAP-hM3D (Gq)-mCherry) in CA1 astrocytes and evaluated the impact of astrocyte activity on excitatory transmission by recording single or very few CA3-CA1 synapses; that is, CA3 Schaffer collaterals (SCs) were electrically stimulated to activate single CA1 hippocampal synapses (see Methods, and Figure 4A,B). Evoked excitatory postsynaptic currents (EPSCs) were recorded in the presence of picrotoxin (50 µM) in basal conditions and after stimulating astrocytes with a local application of CNO (1 mM), both in WT and in Ts65Dn mice. The following synaptic parameters were examined: synaptic efficacy (mean peak amplitude of all responses, including failures); synaptic potency (mean peak amplitude of the successes); and probability of release (Pr, ratio between number of successes versus total number of stimuli).

In WT mice, astrocyte stimulation evoked a synaptic potentiation of EPSCs in 7 of 13 synapses (53.84%) for 5 min upon CNO local application, as evidenced by an increase in synaptic efficacy (paired *t*-test; *p* < 0.05; n = 7 out of 13 astrocyte–neuron pairs from 5 WT mice; Figure 4D, right panel) and probability of neurotransmitter release (Pr) (paired *t*-test; *p* < 0.001; n = 7 out of 13 astrocyte–neuron pairs from 5 WT mice; Figure 4G), while no changes in synaptic potency were detected (paired *t*-test; N.S.; *n =* 7 *out of* 13 *astrocyte–neuron pairs from* 5 *WT mice;* Figure 4I). Conversely, trisomic astrocyte activation by CNO led to a synaptic depression in 42,88% of the synapses (6 of 14) (Figure 4F). Synaptic efficacy (paired *t*-test; *p* < 0.01; n = 6 out of 14 astrocyte–neuron pairs from 4 mice; Figure 4F) and Pr were significantly reduced in Ts65Dn (paired *t*-test; *p* < 0.01; n = 6 out of 14 astrocyte–neuron pairs from 4 mice; Figure 4H), while synaptic potency remained unaltered (paired *t*-test; N.S.; n = 6 out of 14 astrocyte–neuron pairs from 4 mice; Figure 4J).

### 3.4. Glutamatergic, Neuroinflammatory, and Synaptic Transcriptomic Signatures in Trisomic Astrocytes

To understand the possible mechanisms underlying synaptic depression upon astrocyte stimulation, we analyzed from a previously obtained single-nucleus RNA sequencing (snRNA-seq) dataset the transcriptomic alterations of the astrocyte population in Ts65Dn [43]. To do so, we focused on the astrocyte cluster and analyzed 4614 astrocyte nuclei.

**Figure 5:**
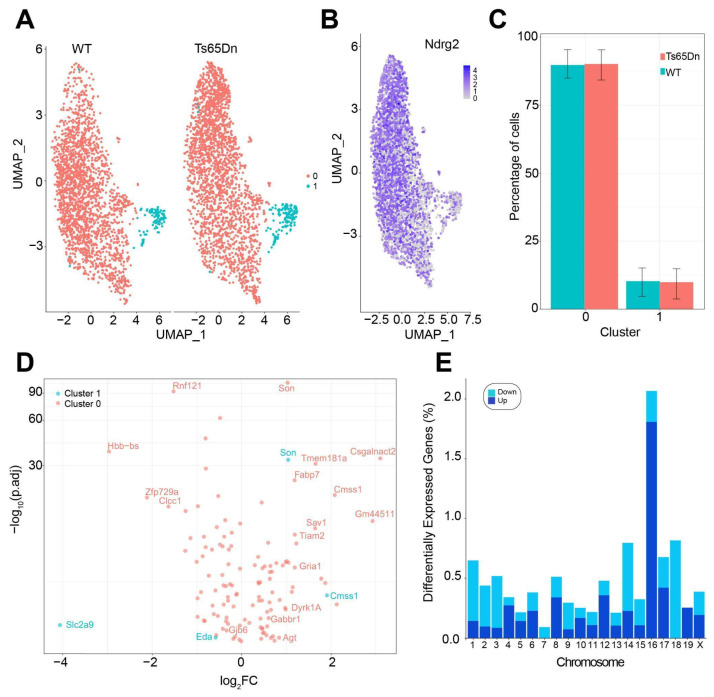
Differential gene expressions in trisomic astrocytes in the hippocampus. (**A**) UMAP projection of single-nucleus RNA sequencing data showing the distribution of astrocytes from WT (**left**) and Ts65Dn (**right**) mice. Two distinct clusters of cells are observed, indicating differences in cell populations. (**B**) Expression of Ndrg2, a marker for astrocytes, visualized on the UMAP plot. High expression levels are shown in darker shades of blue, confirming the identity of astrocytes in the both clusters. (**C**) Bar graph depicting the percentage of cells in each cluster (0 and 1) for WT (blue) and Ts65Dn (red) and astrocytes. (**D**) Volcano plot showing differentially expressed genes between Ts65Dn and WT astrocytes. Genes significantly expressed in Cluster 0 are depicted in red, and those in Cluster 1 are in blue. (**E**) Bar chart representing the percentage of differentially expressed genes (DEGs) across different chromosomes. The chart differentiates between genes that are upregulated (dark blue) and downregulated (cyan) in Ts65Dn astrocytes. Chromosome 16 shows a particularly high proportion of DEGs, reflecting the triplication present in the Ts65Dn mouse model.

By using a Uniform Manifold Approximation and Projection (UMAP) method, we were able to distinguish two main astrocytic populations: mature (Cluster 0), being the main class (94.03% of total astrocytes); and cycling astrocytes (Cluster 1) (Figure 5A), which correspond to previously described AST1 and AST4 astrocyte hippocampal subtypes (Supplementary Figure S4A) [44].

Cluster 1 was enriched in S-phase astrocytes, as measured by the Cell-Cycle Scoring and Regression function using canonical markers [30]. Astroglial markers such as *Ndrg2*, *Gfap*, *S100β*, or *Vimentin* were used to identify astrocyte clusters (Figure 5B). No differences in the percentage of Cluster 0 and Cluster 1 astrocytes were observed between WT and Ts65Dn mice (Figure 5C).

The gene expression analysis revealed a total of 121 DEGs between trisomic and euploid mature astrocytes (Cluster 0), and 4 DEG in cycling astrocytes (Cluster 1; Figure 5D). As expected, most of the upregulated DEG genes were found in the triplicated regions located in Mmu16 (Figure 5E). From the 121 DEG genes in Cluster 0, only 12 were orthologous to the HSA21 (≈7%), suggesting that astrocytes might have a differential susceptibility to the trisomy to gene dosage effects compared to neurons [45], where the percentage of DEGs genes orthologous to HSA21 is around 19% [29] (Supplementary Figure S4B). Triplicated genes involved in cell survival, proliferation, and the gliogenic shift, such as *Dyrk1A*, were significantly upregulated in trisomic astrocytes (Supplementary Table S3). We found an overexpression of *Blbp*, also called *Fabp7,* a marker of reactive astroglia and biomarker of neurotrauma, and angiotensin (*Agt*), which is related with angiogenesis and blood pressure regulation during neuroinflammation (Supplementary Figure S4D,E). *Gjb6*, encoding for connexin 30 (Cx30), was significantly downregulated in trisomic astrocytes. Remarkably, we also found a downregulation of solute carrier family 1 member 2 (*Slc1a2* or *Glt-1*), which is a crucial astrocytic transporter responsible for the reuptake of glutamate from the synaptic cleft, and an upregulation of genes encoding for neurotransmitter receptor subunits of AMPA and GABAB1, such as *Gria1* and *Gabbr1*.

To explore the functional implications of these transcriptional alterations in trisomic astrocytes, we performed a Gene Ontology (GO) enrichment analysis of the DEGs in Cluster 0. This analysis revealed a significant enrichment in processes related to neurodevelopment, cell proliferation, synapse organization, gliogenesis, and neuroinflammation—including pathways such as regulation of membrane potential and glutamate transport (Supplementary Figure S4C). These enriched pathways align well with the functional alterations observed in Ts65Dn astrocytes and may underlie the synaptic deficits described in this model.

## 4. Discussion

In DS, astrocyte dysfunction is detectable early in brain development [9,10]. Previous studies showed an increased astrocyte number; increased astroglial volume; and higher expression of astroglial proteins, such as S100β, both in postmortem DS brains [9,10,11,46,47] and DS mouse models [6,16].

Our findings refine this view, showing that astrocytopathy is not evenly distributed across hippocampal subregions. The CA1 hippocampal subregion, particularly the *stratum oriens* and *stratum lacunosum*, as well as the DG granule cell layer, showed more severe changes, including localized increases in the number astrocytes, and increased astrocyte somatic volumes and S100β expression, suggesting that astrocytopathy may differentially influence the trisynaptic hippocampal circuit. In contrast, CA3 showed no significant increase in astrocyte number, possibly due to its intrinsic recurrent circuitry and a robust mossy fiber input from the DG [48,49], that may buffer the effects of astrocyte dysfunction, maintaining homeostasis despite trisomy-related stressors. Nevertheless, as CA3 function depends on the integrity of downstream and upstream regions, its function is probably hindered by astrocytopathy in the surrounding regions. It is important to consider that although S100β reliably labels most astrocyte somas, it might underestimate subpopulation of astrocytes with low or no S100β expression.

The increased expression of S100β, a Ca^2+^-binding protein located on HSA21 and primarily expressed in astrocytes, was reported to contribute to the spontaneous intracellular Ca^2+^ fluctuations in DS astroglia [15]. This increased S100β was associated with elevated spontaneous Ca^2+^ oscillations in trisomic astrocytes, which were associated with reduced neuronal activity [15]. In contrast, in our experiments, the frequency of spontaneous Ca^2+^ oscillations was not affected in Ts65Dn astrocytes, and the amplitude of the Ca^2+^ transients was significantly higher in trisomic astrocytes. Different mechanisms can contribute to the observed changes in Ca^2+^ oscillation patterns. Elevated basal Ca^2+^ levels can predict the amplitude of Ca^2+^ signals in astrocytes [50], and resting cytosolic Ca^2+^ is elevated in trisomic models such as Ts65Dn mice [51] and in Ts16 mice [52]. The fact that in human iPSC-derived trisomy 21 astrocytes, increased S100β was linked to higher Ca^2+^ oscillation frequency, may be due to the immature developmental stage of iPSCs-derived astrocytes that differ from adult astrocytes in terms of gene expression and calcium dynamics [53,54]. Future studies normalizing candidates that are differentially expressed between WT and trisomic astrocytes and involved in calcium signaling (e.g., *Dyrk1*A and *Gjb6*) or genes directly associated with calcium buffering, such as S100β, will help to dissect their relative contributions to astrocytic Ca^2+^ alterations in Ts65Dn mice. The analysis of calcium dynamics was measured in astrocytes exhibiting spontaneous Ca^2+^ oscillations during the recording time. While this approach allowed us to characterize functional alterations in the active astrocyte subset, it does not capture the behavior of non-oscillating astrocytes. While the number of animals used for calcium imaging was limited (n = 3), the findings demonstrated remarkably consistent patterns across all individuals, with large effect sizes that exceeded biological variability.

The increased amplitude of Ca^2+^ oscillations might lead to atypical modulation of synaptic activity by modifying the release of different gliotransmitters, like ATP, glutamate, or D-serine, disrupting astrocyte–neuron communication. We previously described that selective activation of astrocytes by expressing hM3D (Gq), a designer receptor exclusively activated by designer drugs (DREADD), promoted synaptic potentiation by potentiating transmitter release at single CA3-CA1 hippocampal synapses [1]. In Ts65Dn mice, CNO activation of astrocytes had the opposite effect, inducing synaptic depression, and reducing synaptic efficacy and the probability of neurotransmitter release. Although the mechanism underlying this synaptic depression in Ts65Dn synapses has not been explored in this study, it might be possible that Gq activation in Ts65Dn astrocytes would lead to dysregulated Ca^2+^ responses, resulting in altered or exacerbated gliotransmission by excessive glutamate or ATP, which is metabolized into adenosine and might reduce the probability of glutamate release causing synaptic depression [1]. The differential response of trisomic astrocytes to Gq activation might be due to several factors including elevated resting Ca^2+^ levels, overexpression of Ca^2+^-modulating proteins such as S100β or genetic changes due to the trisomy. However, to what extent these short-term changes in synaptic transmission might translate to hippocampal network alterations in Ts65Dn is difficult to anticipate. Future studies are needed to determine the impact of astrocyte-mediated synaptic modulation on circuit dynamics and behavior in DS.

We then analyzed possible transcriptional alterations leading to trisomic astroglia dysfunction. To this aim, we used previously produced single-nuclei RNAseq data of the different glial subtypes in the hippocampus, including oligodendroglia, astroglia, and microglia, focusing on astrocytes [43]. We identified two clusters of astrocytes: a major cluster of mature astrocytes and a small cluster of cycling astrocytes. This is in line with evidence showing that most astrocytes in the adult brain are post-mitotic and long-lived, whereas the number of newly generated astrocytes is limited [55]. In the snRNA-seq analysis, we did not detect the increase in the number of astrocytes we observed using stereological techniques. This might be attributed to differences in spatial resolution and sampling sensitivity since stereological methods provide precise cell counts in defined subregions, allowing for the detection of localized changes, whereas snRNA-seq of the whole hippocampus might dilute subregion-specific changes.

We found that, from the 121 DEGs genes in the cluster of mature astrocytes, approximately 7% were triplicated genes orthologous to the HSA21. This is a quite reduced number, compared to the impact of trisomy in neurons, where a higher portion of DEGs, mostly upregulated, were located to Mmu16 [29]. Compensatory mechanisms like heterochromatinization might be compensating for the gene overdosage effects in trisomic astrocytes, given the upregulation of *Dyrk1A, Brd9*, and *Arid1b*, along with the downregulation of *Tcf4*, which are involved in chromatin remodeling and transcription regulation [56,57,58,59].

These results suggest that trisomic astrocytes adapt to genetic perturbations but may be less able to adequately respond to environmental stressors, such as inflammation, brain insult, and neurodegeneration [45,60,61,62], as suggested by the downregulation of key genes such as *Clcc1* and *Rnf121* that likely exacerbates Ca^2+^ dysregulation and metabolic dyshomeostasis, while the upregulation of genes like *Fabp7*, *Agt*, and *Sav1* suggests a shift toward a reactive state. Fabp7 is upregulated in astrocytes in response to inflammatory stimuli such as IL-6, and it has been implicated in astrocyte proliferation, neuroinflammation, and glial scar formation following CNS injury. Likewise, Agt expression in astrocytes increases under inflammatory conditions and contributes to local renin–angiotensin signaling, blood–brain-barrier modulation, and immune-cell recruitment. The concurrent upregulation of *Fabp7* and *Agt* suggests that trisomic astroglia are not only more numerous but also exhibit a reactive molecular phenotype. This reactive state might disrupt homeostatic functions, such as supporting synaptic transmission and maintaining the extracellular environment. In line with these results, we observed significant downregulation of *Gjb6*, the gene encoding Connexin 30 (Cx30), in trisomic astrocytes. Unlike neurons, which operate in discrete circuits, astrocytes form an interconnected syncytium via gap junctions composed primarily of Connexin 30 (Cx30) and Connexin 43 (Cx43). The downregulation of Cx30 could disrupt the buffering of extracellular potassium and glutamate [63], as well as activity-induced Ca^2+^ wave propagation, impairing astrocyte-to-astrocyte signaling [64] and being partially responsible for alterations in the calcium dynamics shown in trisomic astrocytes. Interestingly, Cx30 expression is upregulated in response to enriched environments that enhance memory [65]. In contrast, Cx30-deficient mice exhibited behavioral abnormalities, reduced AMPAR-mediated excitatory transmission, impaired synaptic plasticity, and displayed deficits in contextual fear memory [66,67].

We also identified several differentially expressed genes related to synaptic transmission in trisomic astrocytes. Notably, there was an upregulation of genes encoding subunits AMPA and GABAB, such as *Gria1* and *Gabbr1*. Astrocytes expressing AMPA receptors can influence excitatory transmission and influence gliotransmitter release, while GABAB receptor activation regulates inhibitory signaling and Ca^2+^ dynamics [68].

In conclusion, our study reveals region-specific and functional alterations with increased astrocyte number in the CA1 and DG hippocampal subregions, accompanied by an overall increase in the somatic volume and S100β expression, across hippocampal regions. From a functional perspective, we demonstrate a significant increase in the amplitude of spontaneous Ca^2+^ oscillations in CA1, with no changes in the frequency, suggesting deregulated Ca^2+^ dynamics. Importantly, we found that the chemogenetic activation of trisomic astrocytes induced a synaptic depression of CA3-CA1 single synapses, in contrast with the potentiation observed in WT astrocytes, highlighting a shift in the astroglial modulation of synaptic activity in CA1 neurons. These findings highlight that trisomic astrocytes play a crucial role in hippocampal network dysfunction, which may have consequences for the learning and memory impairments shown in Ts65Dn mice. Our results not only expand our understanding of the astrocytopathy associated with DS but also highlight astrocytes as potential targets for therapeutic intervention for restoring synaptic function in DS.

## Data Availability

The data that support the findings of this study are available from the corresponding author upon request.

## Supplementary Materials

The following supporting information can be downloaded at https://www.mdpi.com/article/10.3390/cells14171332/s1, Figure S1: Subregion-specific astrocytopathy in Ts65Dn hippocampus. (A) Neuronal density (Nv) and (B) volume of the somatic layers of CA1, CA3 and DG in the somatic regions of the hippocampus. Gray dots indicate the estimations of every WT mouse while orange dots indicate the estimations of the Ts65Dn mouse. Each dot represents the mean of 6 different sections. Horizontal lines indicate the mean values for every group. (C) Astrocyte density and (D) volume of the dendritic (CA1_Lac, CA1_Or and CA1_Rad) and somatic (CA1_py) layers of the CA1 region. (E) Astrocyte density and (F) volume of the dendritic (CA3_Lac, CA3_Or and CA3_Rad) and somatic (CA3_py) layers of the CA3 region. (G) Astrocyte density and (H) volume of the dendritic (DG_ML, and DG_PL) and somatic (DG_GL) layers of the DG region. On the boxplots, the horizontal line indicates the median, the box indicates the first to third quartile of expression and whiskers indicate 1.5 × the interquartile range. *** *p* < 0.001, * *p* < 0.05. SL = *stratum lacunosum*, SO = *stratum oriens*, SP = *stratum pyramidale*, SR = *stratum radiatum*, GL = *granule layer*, ML = *molecular layer*; Figure S2: Estimation of the astroglial volume. (A) Confocal image showing S100β immunostaining in the CA1 region of the hippocampus (in red). Scale bar = 50 μm. (B) Same image processed to create a binary mask. Astrocytes somas are depicted in white while the background is black. Scale bar = 50 μm. (C–D) Zoomed image of A and B, respectively. Scale bar = 20 μm. (E) Semi-automatic detection of astroglial somatic area in the binary mask image. Scale bar = 20 μm; Figure S3: Ts65Dn astrocytes present a higher amplitude of the spontaneous Ca^2+^ events. (A) Representation of all the spontaneous Ca^2+^ events in WT astrocytes (87 astrocytes from 3 mice). (B) Representation of all the spontaneous Ca^2+^ events in Ts65Dn astrocytes (109 astrocytes from 3 mice). (C) Average trace of WT spontaneous events (blue). Blue shadow indicates the standard deviation of the traces (87 astrocytes from 3 mice). (D) Average trace of Ts65Dn spontaneous events (109 astrocytes from 3 mice); Figure S4: (A) Heatmap showing the Jaccard Index for the overlap between our dataset and the astrocyte subtype markers from the study referenced. The color key indicates the degree of overlap, with significant overlaps marked by their respective *p*-values. Subtypes AST1 and AST4 show significant overlaps with our identified clusters 0 and 1, respectively. (B) Distribution of DEGs in Cluster 0 based on orthology to HSA21. Genes were categorized into two groups: those with orthologs present on human chromosome 21 (HSA21) and those without orthologs on HSA21. Out of the 120 DEGs analyzed, 12 were identified as orthologs to HSA21. (C) Top enriched biological processes based on DEG identified in mature astrocytes. Dot size represents the number of genes associated with each GO term (nGenes), while dot color indicates the false discovery rate (FDR) of enrichment. (D) Mapping of the *Agt* and *Fabp7* both WT and Ts65Dn mice. (E) Violin plots showing single-nucleus RNA expression levels of *Agt* (left) and *Fabp7* (right) in astrocytes from WT and Ts65Dn (TS) mice. Each dot represents an individual nucleus; Table S1: Trisomic astrocyte volume is increased compared to WT littermates in the different subregions of the hippocampus. Volumes of WT and Ts65Dn astrocytes are indicated for every hippocampal subregion. Parentheses indicate the number of astrocytes per region. WT (n = 4 mice), Ts65Dn (n = 4 mice). Mann-Whitney. Data are expressed as mean ± SEM of astrocyte volume (µm^3^). * *p* < 0.05, ** *p* < 0.01, *** *p* < 0.001.; Table S2: Astrocyte S100β expression is increased compared to WT littermates in the different subregions of the hippocampus. Astrocyte S100β expression measured as arbitrary units (a.u.) in the different hippocampal regions in WT and Ts65Dn mice. Background signal subtraction was performed for all the data. Parentheses indicate the number of astrocytes per region. WT (n = 4 mice), Ts65Dn (n = 4 mice). *** *p* < 0.001. Mann-Whitney. Data are expressed as mean ± SEM of S100β fluorescence intensity (a.u.); Table S3: Differentially expressed genes (DEGs) identified in single-nucleus RNA sequencing analysis comparing mature and cycling astrocyte populations. Columns include the adjusted *p*-value (p_val_adj), log2 fold change (avg_log2FC), and percentage of cells expressing the gene in each population (pct.1 for mature astrocytes and pct.2 for cycling astrocytes). The dataset also includes raw *p*-values (p_val), gene identifiers (genes), data source (source), and the logarithm of *p*-values (logp).

## Abbreviations

The following abbreviations are used in this manuscript:

AAV5	Adeno-Associated Virus 5
ACSF	Artificial cerebrospinal fluid
ATP	Adenosine triphosphate
CA1	Cornu Ammonis 1
CA3	Cornu Ammonis 3
CNO	Clozapine N-oxide
CP	Corner point
DEGs	Differentially expressed genes
DG	Dentate gyrus
DREADD	Designed Receptor Exclusively Activated by Designer Drugs
DS	Down syndrome
EPSCs	Excitatory postsynaptic currents
FANS	Fluorescence-Activated Nuclear Sorting
HSA21	Homo Sapiens Autosome 21
iPSCs	Induced pluripotent stem cells
NA	Numerical aperture
NGS	Normal Goat Serum
OD	Optical Disector
PBS	Phosphate-Buffered Saline
PFA	Paraformaldehyde
ROI	Region of interest
SC	Schaffer collaterals
UMAP	Uniform Manifold Approximation and Projection
UMI	Unique Molecular Identifier
WT	Wild type
