# Supplementary material for: Astrocytopathy Is Associated with CA1 Synaptic Dysfunction in a Mouse Model of Down Syndrome
Source: Cells. 2025 Aug 28;14(17):1332. doi: 10.3390/cells14171332 (PMC12428643; doi:10.3390/cells14171332)
Supplement: Supplementary file 1 [file cells-14-01332-s001.zip › cells-3736213-supplementary.pdf]

Supplementary Information

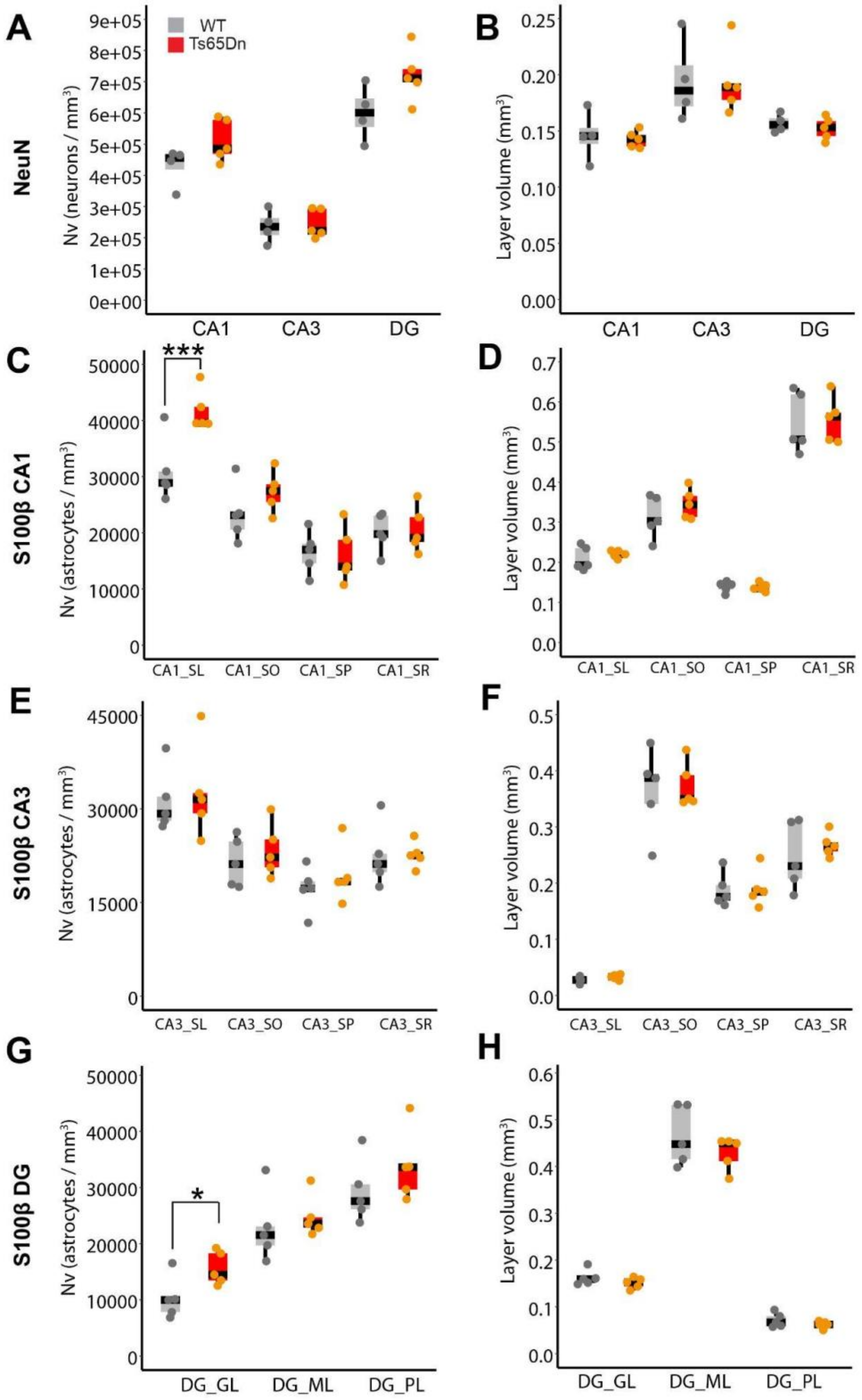

Supplementary Figure S1. Subregion-specific astrocytopathy in Ts65Dn hippocampus. **(A)** Neuronal density (Nv) and **(B)** volume of the somatic layers of CA1, CA3 and DG in the somatic regions of the hippocampus. Gray dots indicate the estimations of every WT mouse while orange dots indicate

the estimations of the Ts65Dn mouse. Each dot represents the mean of 6 different sections. Horizontal lines indicate the mean values for every group. **(C)** Astrocyte density and **(D)** volume of the dendritic (CA1_Lac, CA1_Or and CA1_Rad) and somatic (CA1_py) layers of the CA1 region. **(E)** Astrocyte density and **(F)** volume of the dendritic (CA3_Lac, CA3_Or and CA3_Rad) and somatic (CA3_py) layers of the CA3 region. **(G)** Astrocyte density and **(H)** volume of the dendritic (DG_ML, and DG_PL) and somatic (DG_GL) layers of the DG region. On the boxplots, the horizontal line indicates the median, the box indicates the first to third quartile of expression and whiskers indicate 1.5 × the interquartile range. *** $P < 0.001$, * $p < 0.05$. SL = *stratum lacunosum*, SO = *stratum oriens*, SP = *stratum pyramidale*, SR = *stratum radiatum*, GL = *granule layer*, ML = *molecular layer*.

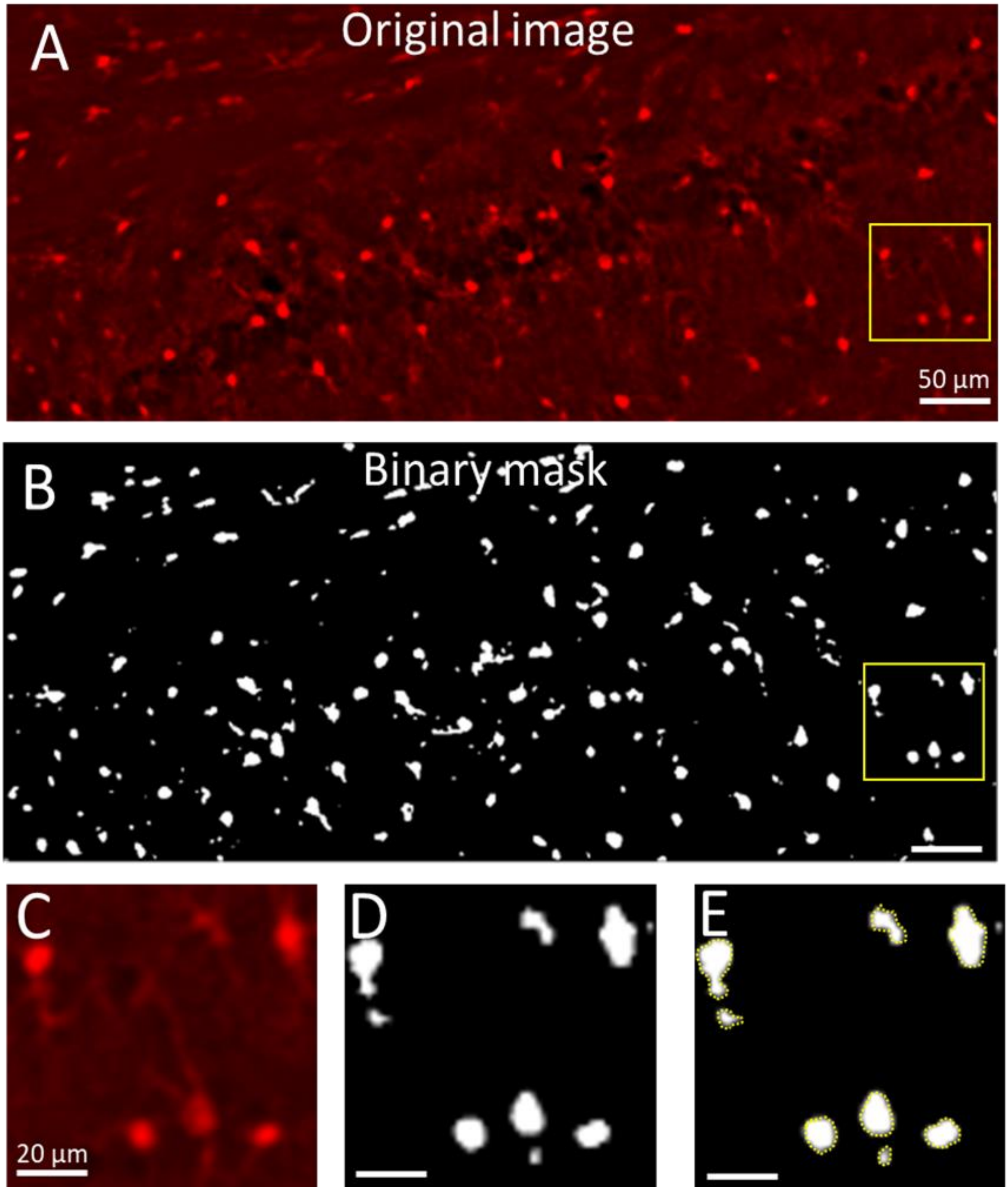

Supplementary Figure S2. Estimation of the astroglial volume. (A) Confocal image showing S100β immunostaining in the CA1 region of the hippocampus (in red). Scale bar = 50 µm. **(B)** Same image processed to create a binary mask. Astrocytes somas are depicted in white while the background is black. Scale bar = 50 µm. **(C-D)** Zoomed image of A and B, respectively. Scale bar = 20 µm. **(E)** Semi-automatic detection of astroglial somatic area in the binary mask image. Scale bar = 20 µm.

Hippocampal region	WT (µm^3)	Ts65Dn (µm^3)	p-value
CA1 lacunosum-moleculare	537,74 ± 24,99 (83)	689,16 ± 37,77 (100) *	0,013
CA1 stratum oriens	697,13 ± 29,97 (106)	883,22 ± 37,79 (103) ***	5,3e-05
CA1 pyramidal layer	630,50 ± 24,74 (87)	817,13± 36,29 (103) ***	7,4e-06
CA1 stratum radiatum	496,86 ± 27,51 (70)	711,81 ±34,20 (80) ***	4,55e-07
CA3 stratum oriens	486,74 ± 23,52 (61)	602,05 ± 21,36 (58) *	0,016
CA3 pyramidal layer	567,24 ± 26,71 (61)	617,52 ± 23,93 (69) ns	0,12
CA3 stratum radiatum + lacunosum	465,17 ± 21,59 (65)	565,91 ± 17,52 (70) *	0,01618
DG granule cell layer	397,46 ± 20,18 (83)	616,72 ± 34,62 (98) ***	2,03e-07
DG molecular layer	649,48 ± 34,85 (75)	785,35 ± 33,05 (98) **	0,0013
DG polymorph layer	397,69 ± 26,78 (76)	577,83 ± 27,29 (88) ***	6,60e-08

Supplementary Table S1. Trisomic astrocyte volume is increased compared to WT littermates in the different subregions of the hippocampus. Volumes of WT and Ts65Dn astrocytes are indicated for every hippocampal subregion. Parentheses indicate the number of astrocytes per region. WT (n = 4 mice), Ts65Dn (n = 4 mice). Mann-Whitney. Data are expressed as mean ± SEM of astrocyte volume (µm^3).
*P < 0.05, **P < 0.01, ***P < 0.001.

Hippocampal region	WT (a.u.)	Ts65Dn (a.u.)	p-value
CA1 lacunosum-moleculare	22689,92 ± 957,37 (92)	32343,01 ± 1120,13 (95) ***	1,3e-08
CA1 stratum oriens	23885,18 ± 977,73 (94)	35872 ± 1001,22 (91) ***	2e-13
CA1 pyramidal layer	26813,90 ± 1317,81 (86)	45498,10 ± 995,90 (95) ***	2e-16
CA1 stratum radiatum	25392,44 ± 1124,57 (90)	38742,31 ± 1055,12 (92) ***	2,2e-13
CA3 stratum oriens	26113,41 ± 886,82 (93)	35011,74 ± 906,14 (97) ***	4,3e-13
CA3 pyramidal layer	30392,78 ± 1142,61 (92)	40352,09 ± 885,00 (93) ***	3,7e-13
CA3 stratum radiatum + lacunosum	24119,93 ± 951,16 (92)	35531,92 ± 1106,62 (93) ***	2e-16
DG granule cell layer	20967,98 ± 1150,94 (81)	30780 ± 1670,08 (81) ***	5,9e-14
DG molecular layer	24771,44 ± 1058,85 (95)	33393,14 ± 1624,42 (87) ***	8,4e-10
DG polymorph layer	23882,27 ± 1101,58 (82)	34304,77 ± 1493,84 (91) ***	5,2e-10

Supplementary Table S2: Astrocyte S100β expression is increased compared to WT littermates in the different subregions of the hippocampus. Astrocyte S100β expression measured as arbitrary units (a.u.) in the different hippocampal regions in WT and Ts65Dn mice. Background signal subtraction was performed for all the data. Parentheses indicate the number of astrocytes per region. WT (n = 4 mice), Ts65Dn (n = 4 mice). ****P* < 0.001. Mann-Whitney. Data are expressed as mean ± SEM of S100β fluorescence intensity (a.u.).

Supplementary Table S3: Differentially expressed genes (DEGs) identified in single-nucleus RNA sequencing analysis comparing mature and cycling astrocyte populations. Columns include the adjusted p-value (p_val_adj), log2 fold change (avg_log2FC), and percentage of cells expressing the gene in each population (pct.1 for mature astrocytes and pct.2 for cycling astrocytes). The dataset also includes raw p-values (p_val), gene identifiers (genes), data source (source), and the logarithm of p-values (logp).

https://docs.google.com/spreadsheets/d/11DbsrBUYODhi7mTYlKC6fRsxOI7EqWJc?rtpof=true&usp=drive_fs

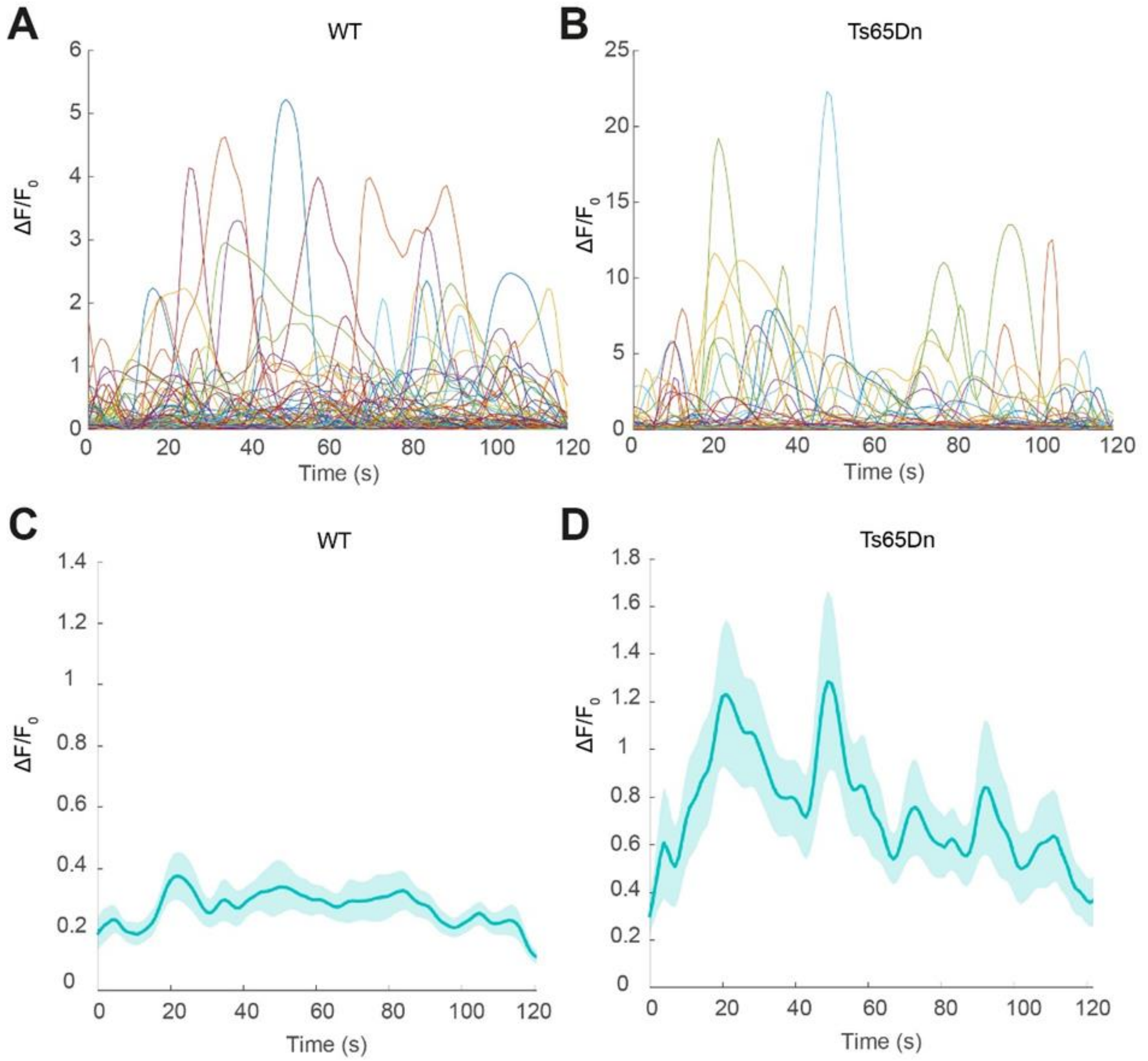

Supplementary Figure S3: Ts65Dn astrocytes present a higher amplitude of the spontaneous Ca^{2+} events. (A) Representation of all the spontaneous Ca^{2+} events in WT astrocytes (87 astrocytes from 3 mice). **(B)** Representation of all the spontaneous Ca^{2+} events in Ts65Dn astrocytes (109 astrocytes from 3 mice). **(C)** Average trace of WT spontaneous events (blue). Blue shadow indicates the standard deviation of the traces (87 astrocytes from 3 mice). **(D)** Average trace of Ts65Dn spontaneous events (109 astrocytes from 3 mice).

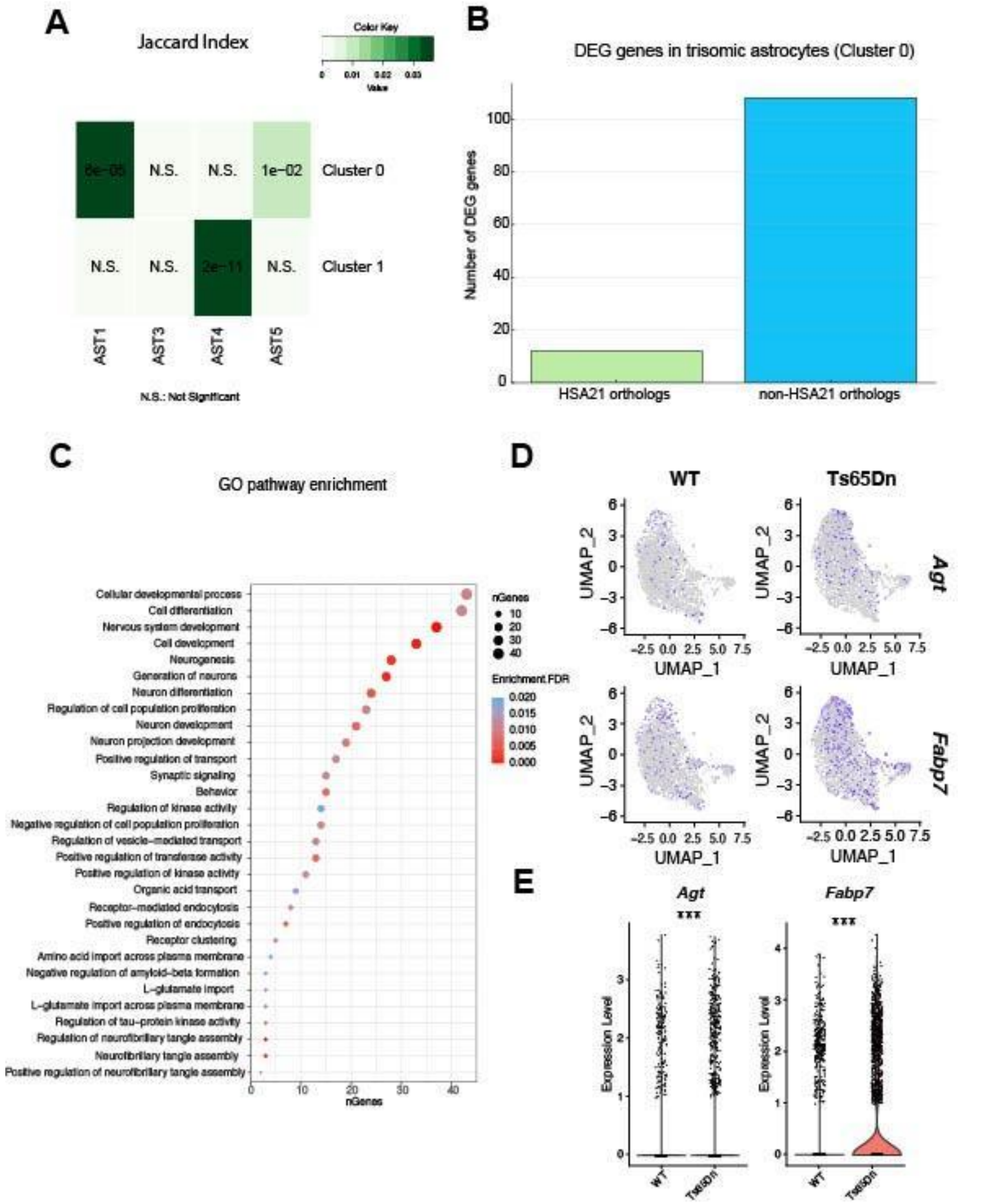

Supplementary Figure S4: **(A)** Heatmap showing the Jaccard Index for the overlap between our dataset and the astrocyte subtype markers from the study referenced. The color key indicates the degree of overlap, with significant overlaps marked by their respective p-values. Subtypes AST1 and AST4 show significant overlaps with our identified clusters 0 and 1, respectively. **(B)** Distribution of DEGs in Cluster 0 based on orthology to HSA21. Genes were categorized into two groups: those with orthologs present on human chromosome 21 (HSA21) and those without orthologs on HSA21. Out of the 120 DEGs analyzed, 12 were identified as orthologs to HSA21. **(C)** Top enriched biological processes based on DEG identified in mature astrocytes. Dot size represents the number of genes associated with each GO term (nGenes), while dot color indicates the false discovery rate (FDR) of enrichment. **(D)** Mapping of

the *Agt* and *Fabp7* both WT and Ts65Dn mice. **(E)** Violin plots showing single-nucleus RNA expression levels of *Agt* (left) and *Fabp7* (right) in astrocytes from WT and Ts65Dn (TS) mice. Each dot represents an individual nucleus.
